# Associations between “Cancer Risk”, “Inflammation” and “Metabolic Syndrome”: A Scoping Review

**DOI:** 10.3390/biology13050352

**Published:** 2024-05-16

**Authors:** Elsa Vitale, Alessandro Rizzo, Kazuki Santa, Emilio Jirillo

**Affiliations:** 1 Scientific Directorate, IRCCS Istituto Tumori “Giovanni Paolo II”, Viale Orazio Flacco 65, 70124 Bari, Italy; 2 Medical Oncology, IRCCS Istituto Tumori “Giovanni Paolo II”, Viale Orazio Flacco 65, 70124 Bari, Italy; a.rizzo@oncologico.bari.it; 3 Faculty of Medical Science, Juntendo University, 6-8-1 Hinode, Urayasu 279-0013, Chiba, Japan; k-santa@juntendo.ac.jp; 4 Scuola di Medicina, University of Bari, 70121 Bari, Italy; emilio.jirillo@uniba.it

**Keywords:** cancer risk, inflammation, metabolic syndrome, oncogenesis, oxidative stress

## Abstract

**Simple Summary:**

Individuals with metabolic syndrome exhibit simultaneously pro-thrombotic and a pro-inflammatory conditions which more probably can lead to cardiovascular disease progression, type 2 diabetes mellitus, and some types of cancer. The present scoping review is aimed at highlighting any relations between cancer risk, inflammation, and metabolic syndrome. A total of 20 manuscripts were identified and, among them, we identified some associations with breast cancer, colorectal cancer, esophageal adenocarcinoma, hepatocellular carcinoma (HCC), and cancer in general. Therefore, it could be deducted that cancer and its related progression may also depend on a latent chronic inflammatory condition associated with other concomitant conditions, including type 2 diabetes mellitus, metabolic syndrome, and obesity. Therefore, prevention may potentially help individuals to protect themselves from cancer.

**Abstract:**

Background: Individuals with metabolic syndrome exhibit simultaneously pro-thrombotic and pro-inflammatory conditions which more probably can lead to cardiovascular diseases progression, type 2 diabetes mellitus, and some types of cancer. The present scoping review is aimed at highlighting the association between cancer risk, inflammation, and metabolic syndrome. Methods: A search strategy was performed, mixing keywords and MeSH terms, such as “Cancer Risk”, “Inflammation”, “Metabolic Syndrome”, “Oncogenesis”, and “Oxidative Stress”, and matching them through Boolean operators. A total of 20 manuscripts were screened for the present study. Among the selected papers, we identified some associations with breast cancer, colorectal cancer, esophageal adenocarcinoma, hepatocellular carcinoma (HCC), and cancer in general. Conclusions: Cancer and its related progression may also depend also on a latent chronic inflammatory condition associated with other concomitant conditions, including type 2 diabetes mellitus, metabolic syndrome, and obesity. Therefore, prevention may potentially help individuals to protect themselves from cancer.

## 1. Introduction

Cancer represents a disease due to dysfunctions and disequilibrium in the DNA balance that advance the malignant development of cells, and most causes of tumors are related to genomic landscapes and genetic aberrations within hematological and solid cancers [1]. Besides genetic etiologies, 44% of cancers may also depend on possible modifiable habits and environmental and occupational risk factors [2], with the consequential increase in incidence of cancers associated with susceptibilities [R2:19], such as metabolic syndrome (MetS), inflammation, and cancer [3]. Cancer is characterized by metastatic development in almost every organ and tissue that may be linked to several etiologic elements, including genomic vulnerability and environmental instability [4]. Cancer growth is also considered a multiphase process in which genetic aberrations grant selected typologies of cancer developments through the progression from physiological cells to malignant ones, likely due to the self-adequacy of growth indications and insensitivity to anti-growth ones, altering apoptosis, proliferation mechanisms, improved angiogenesis, and metastasis [5]. Therefore, in the present scoping review, we aimed to highlight putative associations between cancer risk, inflammation, and metabolic syndrome.

### 1.1. MetS and Cancer Risk

Metabolic syndrome (MetS) is a worldwide health issue, even including a complex pathological condition characterized by the presence of almost three of the following five risk factors: hyperglycemia, hypertension, hypertriglyceridemia, a low level of high-density lipoprotein (HDL) cholesterol, and an increased waist circumference [6]. Individuals with MetS simultaneously may undergo pro-thrombotic and pro-inflammatory conditions, which may lead to cardiovascular disease progression (CVD), type 2 diabetes mellitus (T2DM), and cancer. MetS is often associated with age, obesity and consequential insulin resistance, and lipolysis; the insulin-resistant adipocytes deliver free fatty acids (FFAs) in blood, and visceral obesity, due to a sedentary lifestyle, may release more FFAs in blood circulation as a direct consequence of the modified concentration of adipokines, contrasting and reducing the adiponectin proliferation. The adipokine equilibrium has an impact not only on lipolysis but also on insulin resistance, with an increased production of angiotensinogen and inflammatory cytokines, thus modifying the metabolism of the liver and kidney, ultimately leading to endothelial alteration [7]. The increase in FFAs in the liver induces hypertriglyceridemia that is connected to a reduced cholesterol content in HDL and may lead to hyperglycemia by promoting gluconeogenesis. Lipolysis and insulin resistance also decrease the muscle consumption of glucose, thus contributing to hyperglycemia and promoting hyperinsulinemia. In this regard, the literature also mentions insulin resistance and hyperinsulinemia, as well as insulin-like growth factor-1 (IGF-1), hyperglycemia, and toxic elements being released, even including advanced glycation end products (AGEs) and reactive oxygen species (ROS). All these elements may accelerate oxidative stress and cellular demolition, as well as the release of cytokines and adipokines from adipocytes [8,9,10].

Furthermore, obese adipocytes can cause hypertension through hyperinsulinemia that in turn provokes a raised kidney reabsorption of sodium and urate by increasing the exacerbation of angiotensinogen, with the successive up-regulation of the renin/angiotensin pathway and release of inflammatory cytokines, affecting endothelial dysfunction and oxidative stress with damaged nitric oxide (NO)-dependent vasodilation [11]. Additionally, dyslipidemia may decrease the concentration of high-density lipoprotein cholesterol (HDL-C) and increase the concentration of triglycerides (TGs) and low-density lipoprotein cholesterol (LDL-C) in the plasma [12]. Dyslipidemia has been widely associated with being an essential CVD risk factor. CVDs have further risk factors, such as unhealthy eating behavior, smoking habits, excessive alcohol intake, and sedentary lifestyle [13], and their association with cancer is considered important. Dyslipidemia can also have negative consequences for adaptive immunity by modifying the function and growth of B cells and CD8+ and CD4+ T cells. CD4+ T cells are identified as the most essential supply of the adaptive immune response and are differentiated into several subgroups of Th1 and Th2 cells, follicular helper (Tfh) cells, Th17 cells, and T regulatory (TREG) cells. CD4+ T cells exert several roles, activating both immune and nonimmune cells, cytolytic activity, and, in general terms, modulating the immune response [14]. Additionally, Th17 cells have a different CD4+ effector extraction, playing a protective in host immunity against several pathogens and sustaining different inflammatory conditions. Of note, the differentiation of Th17 cells appears to be closely linked to the differentiation of TREG cells, which instead play an anti-inflammatory role [15,16].

This pathologically adapted mechanism can negatively influence the immune system and its related antitumor role [17]. Thus, modified concentrations of plasma cholesterol can switch to modifications of proteins and redox feedbacks and immune dysregulation by favoring inflammatory cytokine secretion and its consequent endothelial modification, also improving oxidative stress with a reduced nitric oxide (NO)-dependent vasodilation result [18].

### 1.2. Inflammation and Cancer Risk

Inflammation represents the body’s defense to tissue harm, due to physical, ischemic damage, infection, vulnerability to toxins, and other trauma typologies. The body’s inflammatory reaction provokes cellular alterations and immune responses, evoking the repair response of the damaged tissue and cellular growth on the site of the inflammation. If the etiology of the inflammation endures, the inflammatory condition becomes chronic, leading to the failure of some physiological control processes. In the chronic inflammatory condition, cell alteration and growth can generate a microenvironment, which positively induces the development of cancer. Therefore, chronic inflammation has been associated with several oncogenetic phases, such as cellular alteration, advancement, survival, proliferation, invasion, angiogenesis, and metastasis [19]. Additionally, the literature suggests that chronic inflammation may be strictly connected with aging processes, the so-called inflammaging. Several immune cells, ranging from macrophages and neutrophils to eosinophils, are directly involved in proinflammatory cytokine production. The chronic inflammatory microenvironment is mainly made up by macrophages that with other leukocytes secrete great levels of reactive oxygen and nitrogen products [20] during infectious diseases. However, in persistent tissue damage and cellular growth, the infection-fighting agents may play a dangerous role due to the production in DNA-related mutagenic factors which cause alterations in epithelial and stroma cell proliferation; in addition, macrophages and T-lymphocytes may secrete tumor necrosis factor-alpha (TNF-α) and macrophage migration inhibitory factors that are involved in DNA alterations [21]. Moreover, many studies suggest that chronic inflammation can exert a serious role in a wide variety of age-related diseases, including diabetes and cardiovascular and autoimmune diseases. Inflammatory processes induce oxidative stress and reduce cellular antioxidant capacity; moreover, overproduced free radicals reacting with cell membrane fatty acids and proteins seem to permanently impair their functions, too. 

## 2. Materials and Methods

### 2.1. Search Strategy

This scoping review followed the Cochrane guidelines and reported using the Preferred Reporting Items for Scoping Reviews and Meta-analysis (PRISMA) [22]. The protocol research was registered in the Figshare register with doi no.: http://10.6084/m9.figshare.25442860 (created on 20 March 2024).

A search strategy was performed, mixing keywords and MeSH terms, such as “Cancer Risk”, “Inflammation”, “Metabolic Syndrome”, “Oncogenesis” and “Oxidative Stress”, and matching them through Boolean operators (Supplementary File S1).

### 2.2. Inclusion and Exclusion Criteria

The review included all observational study designs, like prospective or retrospective cohort or case-control studies, clinical and randomized controlled trials, reviews, systematic reviews, and meta-analyses, reporting any associations between “Cancer Risk”, “Inflammation”, and “Metabolic Syndrome”. Additionally, systematic reviews, reviews, and meta-analyses were also hand-searched in order to include some additional manuscripts. A manual search of the reference lists of the selected publications was also performed in order to identify additional studies for potential inclusion. Potentially relevant articles were acquired to be full-length texts, and authors were contacted when the article was not available. No time limits were included and only letters to the editor, corrigendum, and expert opinion were excluded. 

### 2.3. Peer Review

Initially, records were identified through a scoping database search and uploaded to a reference management software where duplicate studies were removed. Then, two independent reviewers (E.V. and A.R.) assessed the title and the abstract of the identified studies and unsuitable reports were removed. After that, articles were uploaded, and full texts were assessed more closely for their possible eligibilities. Disagreements about whether a study should be included or not was resolved by discussion and consensus. If the disagreement remained, arbitration from another reviewer was provided. Data collection was extracted by considering the following: study characteristics (author, year of publication, and aim), participants (cancer typology and cluster conditions occurring due to metabolic conditions), and outcome in the highlighted associations between “Cancer Risk”, “Inflammation”, and “Metabolic Syndrome”. 

### 2.4. Total Records

**Figure 1:**
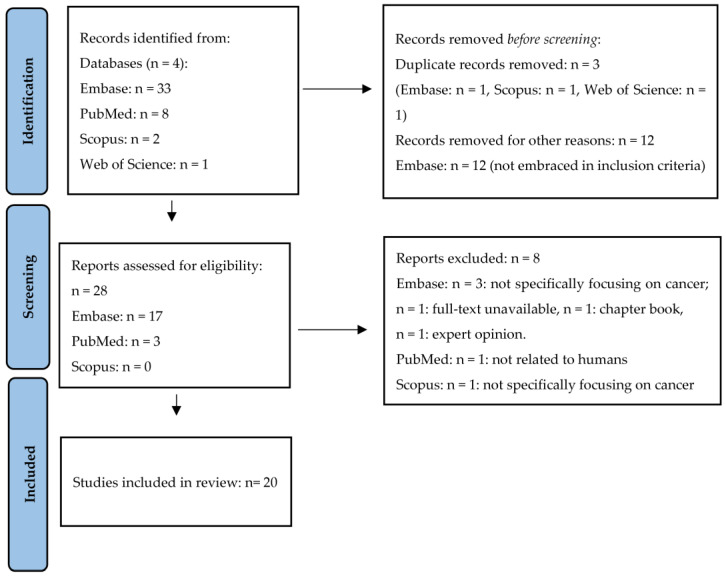
PRISMA flow diagram adopted to the present meta-analysis.

During the first phase of the present scoping review, a total of 44 studies were identified. Of these, 3 studies were removed before screening as they were duplicates and other 12 articles were excluded, as they were not pertinent to our purpose. A total of 28 eligible articles were found. However, 8 of these studies were excluded, since they did not meet the inclusion criteria. Finally, the remaining 20 manuscripts were included in the present scoping review. A summary of the article screening process is presented in the PRISMA flow diagram (Figure 1).

### 2.5. Interventions and Outcomes

The scoping review embraced all typologies of cancer associated with inflammation and metabolic syndrome among adults (≥18 years).

### 2.6. Quality Assessment and Risk of Bias

The studies were assessed for quality as per protocol recommendations. The information retrieved from the final selected studies were exposed using a narrative approach, starting from the oldest to the most recent manuscript to point out the trend of available evidence in this issue over time, too. 

The quality assessment of all the included studies was performed by considering their study designs and related levels of evidence according to the evidence-based nursing (EBN) approach [23]. The EBN strategy embraced a total of seven levels of evidence, ranging from I to VII, suggesting the weakest quality of study design, specifically the following:

- Level I: Evidence from scoping reviews or meta-analysis of randomized control trials;
- Level II: Evidence from well-designed randomized control trials;
- Level III: Evidence from well-designed control trials that are not randomized;
- Level IV: Evidence from case-control or cohort studies;
- Level V: Evidence from scoping reviews of descriptive or qualitative studies;
- Level VI: Evidence from a single descriptive or qualitative study;
- Level VII: Evidence from expert opinions.

In the present scoping review, we included all studies belonging from I to VI levels of evidence.

## 3. Results

From the literature research, a total of 20 articles were selected, which better assess any associations between cancer risk, inflammation, and MetS. All 20 manuscripts belonged to level I of evidence, since they were all scoping reviews. Among the selected papers, there were associations highlighted for breast cancer, colorectal cancer, esophageal adenocarcinoma, hepatocellular carcinoma (HCC), and cancer in general. 

### 3.1. The Association between “Cancer Risk”, “Inflammation”, and “Metabolic Syndrome” in Cancer

A total of nine studies explained the associations between cancer, MetS, and inflammation, without any particular reference to a specific cancer type [24,25,26,27,28,29,30,31,32] (Table 1).

Obesity appears as the most important risk factor in several malignancies, due to several metabolic dysfunctions involved in carcinogenesis. For example, obesity promotes higher estrogens levels, chronic inflammation, and hypoxia, assuming an important function in tumor progression. These processes are connected to a disequilibrium in carbohydrate and lipid metabolism, dysfunctions in the IGF axis and in hormone concentrations, chronic inflammation, and hypoxia. Additionally, increasing BMI and arising insulin and insulin resistance levels represent other cofactors. Besides obesity, diabetes has an impact on cancer risk, too [24]. Additionally, the factors that potentially cause CVDs, diabetes, and cancer cover additional unhealthy lifestyles, such as smoking, obesity, and a sedentary habits. All these risk factors have several pathological mechanisms impacting the increased cancer risk possibility [24,25]. MetS induces chronic low-level inflammation with a constant increase in TNF-α and IL-6 cytokines, especially among obese individuals, and develops insulin resistance and TG circulation. Adipose tissue stimulates local hypoxia and promotes the chronic inflammatory condition involving macrophages and its related release of proinflammatory cytokines, especially TNF-α [28]. FFAs enhance inflammatory signals and trigger NF-kB transcription factors that are essential in both immune response and inflammation. Dead adipocytes secreting FFAs may stimulate toll-like receptor-4 (TLR-4) and the activation of NF-kB with the subsequent release of ROS, pro-inflammatory cytokines, and FFAs, creating a vicious cycle enabling the inflammation process [27]. Therefore, a chronic latent inflammation with a chronic oxidative stress process exposes responsive cells to cancer risk. Also, in T2DM, the metabolism dysfunction provokes a chronic pro-inflammatory condition with a great secretion of pro-inflammatory interleukins (ILs), especially IL-6 and TNF-α, C-reactive protein (CRP), and other chronic inflammatory biomarkers. On the other hand, reactive oxygen species (ROS) favor carcinogenesis by damaging proteins and DNA, with great concentrations of TNF-α and NF-kB, characterizing several multiplications of malignant cells [26,27]. Additionally, cholesterol metabolism modifies immune function in several immunobiologic responses, like T-cell receptors and their related functions in the secretion and activation of neutrophils and macrophages [30]. Conversely, cholesterol plays a role in the wholeness, fluidity, and permeability of membranes, and it has been recognized as an important factor for both cell cycle development and differentiation. Other fundamental regulators of cell development, adhesion, migration, and apoptosis, like mitogen-activated protein kinase (MAPK) and epidermal growth factor receptors, are positioned in lipid rafts [30]. Therefore, an unbalanced physiological equilibrium among HDL-C, LDL-C, and total cholesterol may increase inflammatory factor production, such as TNF-α, IL-6, IL-8, IL-10, and MIP-1 [27,28,30]. At the adipocyte level, insulin decreases lipolysis and inhibits the hormone-sensitive lipase by stimulating lipoproteinlipase (LPL) activity and improving lipogenesis. Hyperglycemia and dyslipidemia may significantly contribute to mutagenesis and carcinogenesis processes and the production of ROS [28]. Hyperglycemia due to hyperinsulinemia may improve the biological activity of insulin-like growth factor (IGF-1), which represents an endocrine and paracrine hormone that regulates tissue development and metabolism [28,32,33]. In diabetic patients, carcinogenesis may be favored by general mechanisms concerning carcinogenesis processes and aggression in other organs since it has been highlighted to be a crucial interaction between hyperglycemia, hyperinsulinemia, peripheral insulin-resistance, and visceral adiposity, something which has been associated with low-grade chronic inflammatory conditions [28,33,34]. In addition, insulin exhibits a mitogenic impact on the processes involved in carcinogenesis and may favor cancer development through IGF-1 [35]. T2DM and obesity may promote additional pathways producing malignant development, with central or visceral adiposity, strongly linked to insulin resistance, in the context of MetS. Therefore, continuous and concomitant low-grade inflammation, insulin resistance, glucose intolerance cytokines (resistin, TNF-α, and IL-6), FFAs, and additional vascular elements released from the visceral adiposity may improve cancer risk and establish a suitable microenvironment for cancer progression. The favorable microenvironment for cancerogenensis is supported by several internal factors, such as oncogenes, gene amplification, and the inactivation of tumor-suppressor genes. On the other hand, external inflammatory or infectious factors may improve the risk of cancer disease [36]. Taken together, these two processes trigger transcription factors in damaged cells, such as NF-κB, STAT-3, and hypoxia-inducible factor 1alpha (HIF-1 alpha) [25]. The transcription elements manage the overexpression, increase the release, or cause the dysfunctional activation of proinflammatory factors, such as cytokines, chemokines, cyclooxygenase-2, prostaglandins, inducible nitric oxide synthase, and nitric oxide. Inflammatory cells, like tumor-infiltrating leukocytes and tumor-associated macrophages (TAMs), have been highlighted in the tumor stroma and supposed as principal mediators in cancer inflammation [25]. The observed inflammatory microenvironment directly encourages tumor growth by arising tumor development due to the acceleration of angiogenesis, invasion, and metastasis processes [25,37]. During the malignant development, the proliferation of cancer cells requires great demands for energy and nutrients to meet the high metabolic requirements. At this phase, cancer cells shift from oxidative phosphorylation to aerobic glycolysis to generate adenosine triphosphate (ATP) [29]. Genome instability plays an important role in the energy metabolism alteration [36], with the altered activation of specific oncogenes, like K-ras [38], MYC [38], mTOR [39], and P53 [40]. Additional somatic changes that may reach the point of causing abnormalities in the mitochondrial genome (mtDNA) have been connected to an increased glycolytic incidence in malignant cells [41]. Moreover, the cancer microenvironment is commonly hypoxic in solid tumors, shifting to the HIF-1α activation by inhibiting mitochondrial respiratory chains and affecting glycolysis. Several typologies of stromal cells, including TAMs, have also been involved in creating a hypoxic cancer microenvironment, thus favoring aerobic glycolysis [42], which shifts to metabolic reprogramming in cancer cells [43]. Glutamine is also involved in cancer cell development by creating an oxidative metabolism starting point, as well as producing the ATP essential for malignant cells [44], and acting as a biosynthetic precursor for several molecules, including FFAs, pyrimidines, purines, and amino acids [44]. 

Moreover, obesity leads to a disequilibrium of IGF and insulin-like growth factor-binding protein (IGFBP) expression [45]. The altered expression of IGFs increases insulin concentration and insulin resistance signaling, which represent the mechanism linked to the development of several cancers. IGF2 also participates in hepatocarcinogenesis to improve neoangiogenesis. Mechanisms implicated in the regulation of IGF impact on the progression of hepatomas through PI3K/AKT and JAK-STAT pathways [45]. The increased expression of IGFBPs may induce dysfunctions in focal adhesion, extracellular matrixes, and structural elements and vice-versa [46]. Since IGFBPs are strictly connected to IGFs and monitor IGF activity, the epigenetic downregulation of IGFBP-4 positively influences cancer growth by decreasing IGF inhibition [47]. Conversely, IGF-independent function is connected to the IGFBP-4 control of cancer development thanks to the estrogen balance of receptor activation [48]. 

### 3.2. The Association between “Cancer Risk”, “Inflammation”, and “Metabolic Syndrome” in Breast Cancer

Two studies explain the association that exists between cancer, MetS, and inflammation in breast cancer [49,50] (Table 2).

As previously stated, obesity increases the risk of cancer and has been associated with chronic disorders. A modified equilibrium in adipokines, especially in leptin, appears to be essential in carcinogenesis mechanisms, cell migration, and metastasis. Leptin favors human epidermal growth factor receptor 2 (HER2) protein concentration through a STAT3-mediated mechanism and the upregulation of the heat shock protein (Hsp90) in breast cancer cells. It has been recognized that insulin and IGFs trigger mitosis in the host and cancerous breast epithelial cells. T2DM and breast cancer may have a positive association [49,51]. In fact, it seems that hyperinsulinemia and eating habits may positively influence energy equilibrium and other hormone dysfunctions, which may be identified as elements involved in the relationship between breast cancer and T2DM [51,52] and obesity and cancer [49,53]. Additionally, the compounded relation between T2DM and cancer may indirectly depend on the same risk factor: obesity [54]. 

### 3.3. The Association between “Cancer Risk”, “Inflammation”, and “Metabolic Syndrome” in Colon Rectal Cancer

One study explains the association between MetS and inflammation and colon rectal cancer (CRC) [55] (Table 3).

Past evidence on CRC and dyslipidemia have just focused on cholesterol, TG, LDL-C, and HDL-C [55]. However, there are different findings with controversial assumptions, as few studies have highlighted an arising risk of CRC with greater TG or cholesterol levels [56,57]. Instead, other reports have focused on the absence of association or negative effect [58], and only very few data linked HDL and LDL with the risk of CRC [59]. 

Cholesterol has an impact on inflammation, which may enhance or hinder apoptosis and cellular production [55]. In fact, hypercholesterolemia is connected with oxidative stress and may contribute to cancer growth [60] by modifying gene delivery, like the adenomatous polyposis coli (APC) gene, which is well identified in balancing cellular production and whose mutation has a key role in the physiological epithelium-adenomatous polyp-malignant neoplasm transformation [61]. Peroxisome proliferator-activated receptor (PPAR) is essential for lipid retention and adipocyte characterization [57]. Evidence suggests the involvement of the PPARγ and the APC gene associated with blood cholesterol and CRC. Moreover, inflammation and serum TG concentrations may be involved in increasing CRC risk [55]. In fact, high TG serum levels may be associated with insulin resistance and obesity, which may induce lipolysis, adiposopathy, and FFA secretion [62,63,64]. Also, this lipolytic process may contribute to the development both of CRC [65] and adipose triglyceride lipase (ATGL), which play a key role in the rate-limiting enzymes involved in lipolysis [55]. TG metabolism is influenced by ATGL through hydrolyzing TG into FFA and diacylglycerol. ATGL-mediated lipolysis liberates an enormous amount of FFA, which is a vital adaptation to the high secretion of tumor cells [65].

### 3.4. The Association between “Cancer Risk”, “Inflammation”, and “Metabolic Syndrome” in Esophageal Adenocarcinoma

Only one study explains the relation that exists between cancer, MetS, and inflammation and esophageal adenocarcinoma (EA) [66] (Table 4).

Evidence associates obesity with an increasing risk of esophageal adenocarcinoma (EA) [67]. A recent meta-analysis [68] suggests a positive association between overweight and obese conditions and EA for both males and females. However, the underlying mechanisms have not been identified so far. Adipose tissue, specifically abdominal and visceral adipose tissue (VAT), has been recognized as metabolically active by releasing substances which are essential for insulin resistance, dyslipidemia, glucose intolerance, hypertension, hypercoagulable conditions, and cardiovascular risk [69]. These altered processes improve EA development by increasing adiposity which may induce a mechanical function in the development of gastro esophageal reflux disease (GERD), through Barrett’s esophagus (BE), and metabolic dysfunctions including pro-inflammatory and pro-cancer activity, which favor dysfunctions in immunological, metabolic, and endocrine systems, especially in male obesity. Moreover, individual factors associated with MetS have been associated with different mechanisms in insulin resistance, aromatase activity, adipokine secretion, angiogenesis, high CRP levels, glucose use, and oxidative stress and its consequent DNA impairment, which together may enhance cancer risk [70]. However, this association is multifactorial, as just mentioned above. Moreover, the obesity–cancer association may also be linked to the endogenous hormone metabolism dysfunctions also including insulin, bio-available sex steroids, IGF-1, and IGFBPs. VAT induces hyperinsulinemia by the secretion of IGFBP1 and 2, increases free insulin and IGF-1, stimulates cell proliferation, inhibits apoptosis, and favors angiogenesis [71]. Increasing adiposity triggers peripheral estrogen secretion from adipose tissue in males and females, and also insulin and IGF-1 hinder sex hormone-binding globulin (SHBG) production. These increases induce the bioavailability of sex steroids, such as androgens and estrogen, and alter cellular differentiation, production, and apoptosis control, favoring the growth of pre-cancer and cancerous cells [71].

### 3.5. The Association between “Cancer Risk”, “Inflammation”, and “Metabolic Syndrome” in HCC

Five studies explain the relation that exists between MetS, inflammation, and hepatic cellular carcinoma (HCC) [72,73,74,75,76] (Table 5).

HCC is an unlucky prognosis, with a high incidence worldwide. Viral infections, alcohol, and steatosis are among the most common HCC risk factors [77]. However, the pathogenesis of this primary liver tumor is more hard to understand and often multifactorial, including genetic mutations in polymorphisms, dysfunctions in metabolic pathways, as in the mitochondrial level, oxidative stress, endoplasmic reticulum (ER) stress, inflammation, and modified secretion in cytokines and adipokines. Most of these molecular dysfunctions have just been suggested in non-alcoholic steatohepatitis (NASH) [78,79]. Alcohol-related liver disease (ALD) and non-alcoholic fatty liver disease (NAFLD) appear to have overlapped pathogenetic aspects in hepatic damage and in the progression of HCC. NASH is the first recognized inflammatory subgroup of non-alcoholic fatty liver disease (NAFLD), associated with steatosis and hepatocyte damage, like ballooning and lobular inflammation, with or without fibrosis [74,80]. The presence of insulin resistance and T2DM has been recognized as a risk factor for several diseases in NAFLD, and also in normal alanine aminotransferase (ALT) conditions [81]. T2DM seems to have an important role in the hepatocarcinogenesis process, especially in patients with NAFLD [74]. In HCV patients with cirrhosis or hepatic steatosis [82], T2DM is strictly associated with an increased risk of HCC. The metabolic dysfunction-associated steatotic liver disease (MASLD) is expressed by exaggerated lipid storage with insulin resistance. Several reports have studied MASLD and MetS and have identified the action of insulin resistance/T2DM, obesity, hypertension, and dyslipidaemia [83] by identifying MASLD as a multifactorial and system-related disorder, which may progress to metabolic dysfunction-associated steatohepatitis (MASH), cirrhosis, and mortality [84,85]. Additionally, MASLD may be caused by environmental factors, like lifestyle, gut microbiome, dietary choices, and obesity, which lead to lipid accumulation, insulin resistance, and dysfunctions in the gut microbiota [86], with an unbalanced adipose tissue lipolysis and an increase in FFA release to the liver with consequent hepatic fat storage. The consequent lipotoxicity provides mitochondrial alteration, with the release of reactive oxygen types and endoplasmic reticulum stress and insulin resistance and lipopolysaccharide absorption from the dysfunctional gut microbiome. Insulin resistance contributes to adipokine and proinflammatory cytokine production [85]. Cellular injury amplifies signals of immune cell infiltration, fibrogenesis, and consequent hepatic progenitor cell activation. 

Both obesity and T2DM maybe arise the incidence of NASH and its related complexity, like cirrhosis and HCC [80]. Most comorbidities have been connected to T2DM and obesity. Obesity has also been connected to hypertension and the high frequency of myocardial infarction and apoplectic stroke [87]. Exaggerated hepatic fat storage in obese individuals is strictly associated with insulin resistance, diabetes, and dyslipidemia, thus supporting the consequential NASH and HCC disease [78]. 

Obesity, together with insulin resistance, has been recognized as the main inducing factor for the progression of liver steatosis [80,88,89], with its related elements of steatosis, such as the innate and adaptive immune functions, insulin resistance, inflammation, and fibrosis during NASH development [90]. In the first phase of inflammation linked to NASH, both macrophages and lymphocytes are the most common inflammatory infiltrates in liver tissue [76]. In the NASH condition, the ROS secretion and its related pro-inflammatory cytokine production (IL-1 and IL-18 in macrophages) appear altered [76]. Macrophages (M1) release pro-inflammatory and immunostimulatory cytokines (IL-1, TNF-α, INF-1), which contribute to antigen performance, due to an anti-tumorigenic function [76]. Moreover, macrophages (M2) activated by IL-4 or IL-13 release anti-inflammatory and immunosuppressive functions, which induces pro-carcinogenesis implications [76]. Tumor cells in HCC can influence macrophages to become M2-TAM, which are involved in the deterioration of cytotoxic T cells (CD8+ T cells) in tumor tissues [76]. TNF receptor-member 4 (TNFRSF4, also known as OX40) hinders Th1 and Th17 differentiation and suppresses monocyte migration, antigen presentation, and M1 polarization. TNFRSF4 levels are positively correlated to NASH, with an increasing level of the Th17/Treg cell ratio [76]. As a consequence of NASH, natural killer T (NKT) cells show high levels of IL-12, type I interferons, and IL-1 [76]. NKT cells and CD8+ T cells enhance liver injury with the secretion of IFNα, generating a promoting microenvironment and altering NASH hepatocytes.

### 3.6. The Association between “Cancer Risk”, “Inflammation” and “Metabolic Syndrome” in Prostate Cancer

Considering prostate cancer, only one report studied the relation between cancer, MetS, and inflammation in prostate cancer [91] (Table 6).

Prostate cancer is among the most common cancer worldwide, with namely 2.6 million of new cases each year [92], and the main risk factors are family history, race, and age [93]. Researchers highlighted associations between geographic zones and prostate cancer incidence by suggesting that Western lifestyles, especially nutrition habits, may represent an important risk factor in its etiology [93]. However, further associations have been displayed between MetS and prostate cancer risk [94]. MetS involves insulin resistance with high insulin concentrations, which incentivize lipogenesis, steroidogenesis, and protein production, and, as a growth factor element, incentivizes cellular production with anti-apoptotic roles, especially in hormone-independent prostate cancer cells [95]. Therefore, MetS may represent an essential prognostic element in prostate cancer strictly linked to CVDs and castration-resistant prostate cancer [96], PSA recurrence, and metastases [97]. Evidence suggests the existence of a linkage between inflammation, prostate cancer, and the chronic inflammation of the prostate gland, which may be a common symptomatology conducible to MetS patients, also associated with high levels of cytokines, interleukins, and growth factors, such as IL-6, MSR1, TNFα, and IL-8 [91], which induce prostate cell differentiation and storage in mutations and epigenetic point modifications [88]. Additionally, VAT may promote inflammatory microenvironment, particularly in MetS patients, also characterized by immune cells gathering [91]. In fact, prostate cancer patients suffering from MetS also report high serum leptin concentrations and low adiponectin levels [98], which together are associated with prostate cancer induction and growth. Leptin favors the same pathway of insulin and IGF-1 and independently increases prostate cell hormones [91]. Additionally, insulin and IGF-1, thanks to the IRS-1-PKB -FoX01 pathway, growth factors via the Ras-ERK-NFATc4 pathway, IL6 and IL-8 via the STAT3-cFOS-FoX01 axis, and FFAs induce an important inhibition of adiponectin gene expression, which is vital in prostate cancer prevention and handling due to its related antiangiogenic activity, and it is also inhibited via the AMPK-TSC pathway, resulting in the prevention of mTOR activation [98]. 

## 4. Conclusions

The literature suggests important associations between cancer risk, inflammation, and MetS. In this respect, dietary prevention may potentially help individuals to protect themselves from malignancies. In fact, modified adipocytes and their related tissue-resident immune cells favor the secretion of inflammatory adipokines and cytokines (IL-1b, IL-2, IL-6, Il-12, and TNF-α). At the same time, oxidative stress represents an essential element in the progression of diseases, like CVDs, neurodegenerative diseases, diabetes, and cancers. Therefore, diet may positively contribute to chronic inflammation prevention and may also result in a better microenvironment creation.

Some characteristics, such as gender and age, may inevitably influence the incidence of the abovementioned association. It has been seen how MetS, obesity, and T2DM are linked with altered estrogen secretions. In prostate cancer, dietary habits are also important in the incidence of the disease. Therefore, nutrition plays a key role in both counteracting obesity, MetS, and the subsequent secretion of pro-inflammatory cytokines. In addition, age plays an essential role as free radicals may lead to mutations and DNA damage that may represent a predisposing factor for cancer and age-related disorders. However, further studies are necessary to properly consider antioxidants as preventive agents, especially for cancer and aging processes [99].

## Figures and Tables

**Table 1:** Associations between “Cancer Risk”, “Inflammation”, and “Metabolic Syndrome” in cancer.

Author(s) Publication Year Level of Evidence	Oncogenesis or Cancer Typology	Biomarkers Assessed	Conditions Assessed	Findings
Jee et al., 2005 [24] level I	Cervix Colon/Rectum Breast Esophagus Leukemia Liver Pancreas Stomach	AP-1 CRP FFA IGFBP3 IGF1 IL-6 IL-1β Insulin MCP-1 MMP-9 NF-κB PAI-1 TF TNF-α	Central obesity Dyslipidemia, hyperglycemia Hypertension	A high BMI increased the risk of colon cancer but was not associated with rectal cancer. Triglyceride levels in the blood did not increase the risk of colorectal cancer.
Sumantran and Tillu 2012 [25] level I	Oncogenesis	Ama COX-2 HIF-1 alpha IL-6 iNOS MCP-1 NF-κB NO OLR-1 PPARs Prostaglandins STAT-3 TNF-α	Abnormal lipid metabolism Chronic inflammation Diet Obesity T2DM	Tumor-infiltrating leukocytes and TAMs were recognized in the tumor stroma. The inflammatory microenvironment directly improved tumor progression, evasion, of apoptosis, and accelerated the processes of angiogenesis, invasion, and metastasis.
Gristina et al., 2015 [20] level I	CRC HCC	C-Reactive IGF1 IL-6 PI3K TNF-α	BMI Hyperinsulinemia Obesity T2DM	T2DM and MetS were directly associated to obesity-related hyperinsulinemia and increasing levels of IGF-1. These mechanisms were considered as key factors in carcinogenesis.
Veniou et al., 2016 [27] level I	Bladder Breast Colon Colorectal Endometrium Gastric HCC Lung Ovary Pancreas Prostate Rectal Thyroid	Adiponectin AMPK IGF-1 IGFBP-3 IR HDLc Leptin mTOR Triglycerides	Aromatase BMI Cytotoxic products inducing insulin resistance Dysglycemia Hyperinsulinemia Hypertension Inflammation MetS Obesity	Insulin stimulated the production of IGF-1 by upregulating the GH receptors in the liver. Activation of IGF-1R stimulated cell proliferation through RAS/MAPK signaling pathway with anti-apoptotic consequence via the PI3K/AKT pathway. Among patients with wild-type RAS cancers, the prognosis was dismal in the obese subgroup.
Battelli et al., 2019 [28] level I	Oncogenesis	COX-2 HIF-1α Insulin LDL NF-kβ NO ROS Uric Acid VEGF XOR	Inflammation MetS Oncogenesis Oxidative stress T2DM	XOR was involved in the pathogenesis of both MetS and cancer through the inflammatory response and the oxidative stress. ROS and nitrogen species and the uric acid derived from XOR improved hypertension, dyslipidemia and insulin resistance, participating in cell transformation and proliferation and also in the progression and metastasis process.
Yu et al., 2020 [29] level I	General	GDH1 K-ras mTOR MAPK MYC P53 Statins TNF-α	Cell signaling pathways Chronic inflammation Dyslipidemia Hyperglycemia Inflammation MetS Obesity Oxidative stress ROS	Increased ROS production, chronic inflammation, and aberrant activation of oncogenic signaling pathways represent important links between metabolic disorders and cancer.
Neshat et al., 2022 [30] level I	Breast Cervical Colon Endometrial Epithelial Esophageal General Gastric Hematological Liver Lung Ovarian Prostate	HDL-C LDL-C Statins TG	CVD Dyslipidemia Hypercholesterolemia	Cholesterol, HDL-C, LDL-C, and TG levels and statins could positively impact on the incidence, progression, and prognosis of different types of cancer, such as lung, prostate, ovary, breast, and gastrointestinal cancers.
Sergeeva et al., 2023 [31] level I	Adrenocortical Breast Colon CRC Endometrium Esophageal HCC Melanoma	Estrogens HbA1c IGFBP IGFBPL1 MAPK mTOR PI3K	Chronic inflammation Hyperinsulinemia Hypoxia Inflammation Lipid metabolism Obesity Oxidative stress	Obesity and cancer development was based on several alterations of metabolism. Increased levels of glucose, fructose, and lipids could be linked to increased food uptake with altered expression of factors regulating metabolic processes under obesity. Obesity was associated with IGF axis alterations and increased estrogen levels. Low-grade chronic inflammation, deregulation of adipokines levels, and hypoxia associated to obesity were very important in cancer genesis and its progression.
Pandit et al., 2024 [32] level I	CRC General	AMPK FFAs HER2 Hsp90 IGFs IL-1 IL-6 IL-8 NF-kβ STAT3 TNF-α	BMI Leptins STA-3-mediated	Higher BMI scores was associated to increased risks of malignancies.

Abbreviations: AMPK: adenosine monophosphate-activated protein kinase is an enzyme; AP-1: activating protein-1; COX-2: cyclooxygenase-2; CRC: colorectal cancer; CRP: C-reactive protein; CVD: cardiovascular disease; GDH1: glutamate dehydrogenase; HbA1C: glycated hemoglobin; HCC: hepatocellular carcinoma; HDL-C: total cholesterol, high-density lipoprotein cholesterol; HER2: human epidermal growth factor receptor 2; HIF-1 alpha: hypoxia-inducible factor 1alpha; Hsp90: heat shock protein; IGF-1: insulin-like growth factor-1; IGFBP3: insulin-like growth factor binding protein; IL-6: interleukine-6; iNOS: inducible nitric oxide synthase; IR: insulin receptor; LDL-C: low-density lipoprotein cholesterol; MCP-1: monocyte chemotactic protein-1; MMP-9: matrix metalloproteinase-9; mTOR: mechanistic target of rapamycin; NF-κB: factor-kappa B; NO: nitric oxide; OLR-1: oxidized LDL receptor 1, PAI-1: plasminogen activator inhibitor-1; PI3K: phosphoinositide 3-kinase; PPARs: peroxisome proliferator-activated receptors; ROS: reactive oxygen species; STAT: signal transducer and activator of transcription; TF: tissue factor; TG: triglyceride; T2DM: type 2 diabetes mellitus; VEGF, vascular endothelial growth factor; XOR: xanthine oxidoreductase.

**Table 2:** Associations between “Cancer Risk”, “Inflammation”, and “Metabolic Syndrome” in breast cancer.

Author(s) Publication Year Level of Evidence	Biomarkers Assessed	Conditions Assessed	Findings
Crujeiras et al., 2013 [49] level II	HIFIα IGFs IL-1β IL-6 IL-8 NF-κ B TNF-α STAT3	Obesity Oxidative stress T2DM	Obesity-related oxidative stress could be a direct cause of neoplastic transformation associated with obesity and T2DM in breast cancer cells.
Iacoviello et al., 2021 [50] level I	CRP NF-κ B PAI-1 t-PA TF u-PA	Environmental Inflammation Lifestyle Reproductive factors	Breast cancer was a typically hormone-dependent tumor and was affected from the susceptibility to common pathogenetic triggers and intermediate phenotypes (T2DM).

Abbreviation: CRP: C-reactive protein; IL: interleukine; PAI-1: plasminogen activator inhibitor 1; T2DM: type 2 diabetes mellitus; t-PA: tissue-type; TNF-α: tumor necrosis factor-α; u-PA: urokinase-type plasminogen activator.

**Table 3:** Associations between “Cancer Risk”, “Inflammation”, and “Metabolic Syndrome” in colon rectal cancer.

Author(s) Publication Year Level of Evidence	Biomarkers Assessed	Conditions Assessed	Findings
Hsu et al., 2022 [55] level I	HDL LDL TG	BMI Hyperglycemia, hyperlipidemia Hypertension MetS T2DM	Patients with DM should monitor TG and cholesterol level through diet, exercise, or taking medications more aggressively, not only for preventing cardiovascular disease but also for first prevention of CRC.

Abbreviations: CRC: colorectal cancer; DM: diabetes mellitus; LDL: low-density lipoprotein; TG: triglyceride.

**Table 4:** Association between “Cancer Risk”, “Inflammation”, and “Metabolic Syndrome” in esophageal adenocarcinoma.

Author(s) Publication Year Level of Evidence	Biomarkers Assessed	Conditions Assessed	Findings
Rayan et al., 2011 [66] level I	Adiponectin Adipsin Angiotensinogen Cholesterol transfer protein FFAs Galectin-12 IL-6 Lactate Leptin Lipoprotein lipase Monobutyrin Phospholipid transfer plasminogen activator inhibitor-1 Prostaciclin Protein Prostaglandin Resistin TNF-α	BMI Obesity	Obesity was positively associated with EA. The systemic inflammatory condition induced the production of adipocytokines and pro-coagulant factors released by adipocytes.

Abbreviations: BMI: body mass index; EA: esophageal adenocarcinoma; FFAs: free fatty acids; IL-6: interleukine-6; TNF-α: tumor necrosis factor-α.

**Table 5:** Associations between “Cancer Risk”, “Inflammation”, and “Metabolic Syndrome” in HCC.

Author(s) Publication Year Level of Evidence	Biomarkers Assessed	Conditions Assessed	Findings
Pocha et al., 2019 [72] level I	DAMP mTOR NF-kβ PAMOs ROS SCGA TLR TMA TNF-α VEGF	ALD BMI HBV HCV IR MetS NAFLD	In diabetic patients, heavy alcohol use of greater than 80 g/day increased the risk of HCC from 2.4 to 9.9. Obesity also had a synergistically effect.
Gutiérrez-Cuevas 2022 [73] level I		MetS Obesity Insulin resistance T2DM	NASH and HCC were closely associated with obesity and diabetes, including metabolic syndrome and NAFLD.
Montesi et al., 2013 [74] level I	ChREBP FFA G6PC GCKR GLP-1 LDL LPL MAP-K PI3-K	Hyperinsulinemia Insulin resistance NAFLD Obesity T2DM	Insulin resistance (reduced flux of PI3-K) was followed by compensatory hyperinsulinemia, as stimulus for carcinogenesis, along the MAP-K pathway and adaptive mechanisms chronically maintained in an obesiogenic environment favored oxidative stress and inflammation, improving the immune responses and increasing the carcinogenic potential.
Nakatsuka and Tateishi 2023 [75] level I	AKT HCV IGF-1 IL-6 NF-kβ PI3K mTOR ROS TNF-α	Chronic inflammation DNA damage Hepatocarcinogenesis, hyperinsulinemia IR Obesity Oxidative stress NAFLD Steatosis T2DM	More than 50% of cases of NAFLD-caused HCC, with a strong association with MetS and T2DM, may be due to its unique nature of increasing lipotoxicity-mediated chronic inflammation.
Phoolchund and Khakoo 2024 [76] level I	Genetic polymorphisms ROS	Inflammation MASDL MASH NAFLD Obesity	MASLD-HCC could develop at an earlier phase of fibrosis.

Abbreviations: ALD: alcohol-related liver disease; ChREBP: carbohydrate response element binding; DAMP: damage-associated molecular patterns; GCKR: glucokinase regulatory protein; G6PC: glucose 6-phosphatase; HCC: hepatocellular carcinoma; HBV: hepatitis B virus; HCV: hepatitis C virus; IGF-1: insulin-like growth factor-1; IR: insulin resistance; MASDL: metabolic dysfunction-associated steatotic liver disease; MASH: metabolic dysfunction associated steatohepatitis; MetS: metabolic syndrome; NAFLD: non-alcoholic fatty liver disease; NASH: non-alcoholic steatohepatitis; PI3-K: phosphatidyl-inositol-3-kinase; ROS: reactive oxygen species; TMA: trimethylamine; T2DM: type 2 diabetes mellitus; TNF-α: tumor necrosis factor-α; TLR: toll-like receptor.

**Table 6:** Associations between “Cancer Risk”, “Inflammation”, and “Metabolic Syndrome” in prostate cancer.

Author(s) Publication Year Level of Evidence	Biomarkers Assessed	Conditions Assessed	Findings
Quagliarello et al., 2017 [91] level I	Adiponectin levels IGF-1 Leptin	Endocrine disruptors Nutrition	Chronic inflammation of prostate gland, which was common in patients affected by MetS, was associated with a higher concentration of cytokines, interleukins, and growth factors that induced prostate cell division with accumulating point mutations and epigenetic modification like DNA hypermethylation of caretaker genes GSTP1, RASSF1A, and APC.

Abbreviations: IGF-1: insulin-like growth factor-1; MetS: metabolic syndrome.

## Data Availability

Data are available upon reasonable request to the corresponding author.

## Supplementary Materials

The following supporting information can be downloaded at: https://www.mdpi.com/article/10.3390/biology13050352/s1, File S1: Search strings carried out to perform this systematic and meta-analysis study.
