# Supplementary material for: Associations between “Cancer Risk”, “Inflammation” and “Metabolic Syndrome”: A Scoping Review
Source: Biology (Basel). 2024 May 16;13(5):352. doi: 10.3390/biology13050352 (PMC11117847; doi:10.3390/biology13050352)
Supplement: Supplementary file 1 [file biology-13-00352-s001.zip › biology-2975573-supplementary.pdf]

Supplementary File S1. Search strings carried out for this scoping review.

Database	Results
**PubMed**<br>Search: ((((Cancer risk) AND (Inflammation)) AND (Metabolic syndrome)) AND (Oncogenesis)) AND (Oxidative stress)<br>("cancer s"[All Fields] OR "cancerated"[All Fields] OR "canceration"[All Fields] OR "cancerization"[All Fields] OR "cancerized"[All Fields] OR "cancerous"[All Fields] OR "neoplasms"[MeSH Terms] OR "neoplasms"[All Fields] OR "cancer"[All Fields] OR "cancers"[All Fields]) AND ("risk"[MeSH Terms] OR "risk"[All Fields]) AND ("inflammation"[MeSH Terms] OR "inflammation"[All Fields] OR "inflammations"[All Fields] OR "inflammation s"[All Fields]) AND ("metabolic syndrome"[MeSH Terms] OR ("metabolic"[All Fields] AND "syndrome"[All Fields]) OR "metabolic syndrome"[All Fields]) AND ("carcinogenesis"[MeSH Terms] OR "carcinogenesis"[All Fields] OR "oncogenesis"[All Fields]) AND ("oxidative stress"[MeSH Terms] OR ("oxidative"[All Fields] AND "stress"[All Fields]) OR "oxidative stress"[All Fields])<br>Translations<br>Cancer: "cancer's"[All Fields] OR "cancerated"[All Fields] OR "canceration"[All Fields] OR "cancerization"[All Fields] OR "cancerized"[All Fields] OR "cancerous"[All Fields] OR "neoplasms"[MeSH Terms] OR "neoplasms"[All Fields] OR "cancer"[All Fields] OR "cancers"[All Fields]<br>risk: "risk"[MeSH Terms] OR "risk"[All Fields]<br>Inflammation: "inflammation"[MeSH Terms] OR "inflammation"[All Fields] OR "inflammations"[All Fields] OR "inflammation's"[All Fields]<br>Metabolic syndrome: "metabolic syndrome"[MeSH Terms] OR ("metabolic"[All Fields] AND "syndrome"[All Fields]) OR "metabolic syndrome"[All Fields]<br>Oncogenesis: "carcinogenesis"[MeSH Terms] OR "carcinogenesis"[All Fields] OR "oncogenesis"[All Fields]<br>Oxidative stress: "oxidative stress"[MeSH Terms] OR ("oxidative"[All Fields] AND "stress"[All Fields]) OR "oxidative stress"[All Fields]	8
**EMBASE**<br>('cancer risk'/exp OR 'cancer risk') AND ('inflammation'/exp OR 'inflammation') AND ('metabolic syndrome'/exp OR 'metabolic syndrome') AND ('oncogenesis'/exp OR 'oncogenesis') AND ('oxidative stress'/exp OR 'oxidative stress')	33
**Scopus**<br>“Cancer Risk” AND “Inflammation” AND “Metabolic syndrome” AND “Oncogenesis” AND “Oxidative Stress”	2
**Web of Science**<br>cancer risk (All Fields) and inflammation (All Fields) and metabolic syndrome (All Fields) and oncogenesis (All Fields) and oxidative stress (All Fields)	1
